# Synthetic bovine lactoferrin peptide Lfampin kills *Entamoeba histolytica* trophozoites by necrosis and resolves amoebic intracecal infection in mice

**DOI:** 10.1042/BSR20180850

**Published:** 2019-01-08

**Authors:** César Díaz-Godínez, Ximena González-Galindo, Thuluz Meza-Menchaca, Raúl J. Bobes, Mireya de la Garza, Nidia León-Sicairos, Juan P. Laclette, Julio C. Carrero

**Affiliations:** 1 Department of Immunology, Instituto de Investigaciones Biomédicas, Universidad Nacional Autónoma de México, 04510, México, D.F., México; 2 Laboratorio de Genómica Humana, Facultad de Medicina, Universidad Veracruzana, Calle Médicos y Odontólogos S/N., Col. Unidad del Bosque, 91010, Xalapa, Veracruz, México; 3 Department of Cellular Biology, Centro de Investigación y de Estudios Avanzados del IPN, Av. IPN 2508, San Pedro Zacatenco, 07360 México, D.F., México; 4 CIASaP, Facultad de Medicina, Universidad Autónoma de Sinaloa, Cedros y Sauces, Fracc. Fresnos Culiacán, 80246, Sinaloa, México; 5 Departamento f Research, Hospital Pediátrico de Sinaloa, Boulevard Constitución S/N, Col. Jorge Almada, Culiacan, 80200, Sinaloa, México

**Keywords:** Amoebicidal activity, Internalization, Intestinal protection, Lactoferrin, Lactoferrampin, Necrosis

## Abstract

Amoebiasis caused by the protozoan parasite *Entamoeba histolytica* remains a public health problem in developing countries, making the identification of new anti-amoebic compounds a continuing priority. Previously, we have shown that lactoferrin (Lf) and several Lf-derived peptides exhibit *in vitro* anti-amoebic activity independently of their iron-binding activity. Here, we evaluated the amoebicidal effect of synthetic Lf-derived peptides Lfcin-B, Lfcin 17-30, and Lfampin, analyzed the mechanism of death induced by the peptides and determined their therapeutic effects on murine intestinal amoebiasis. MTT assays in trophozoite cultures of *E. histolytica* exposed to each peptide (1–1000 μM) showed that Lfampin is far more amoebicidal than Lfcins. Lfampin killed 80% of trophozoites at doses higher than 100 μM in 24 h, and FACs analysis using Annexin V/propidium iodide showed that death occurred mainly by necrosis. In contrast, Lfcin-B and Lfcin 17-30 appeared to have no significant effect on amoebic viability. FACs and confocal microscopy analysis using FITC-labeled peptides showed that all three peptides are internalized by the amoeba mainly using receptor (PI3K signaling) and actin-dependent pathways but independent of clathrin. Docking studies identified cholesterol in the amoeba’s plasma membrane as a possible target of Lfampin. Oral treatment of intracecally infected mice with the abovementioned peptides at 10 mg/kg for 4 days showed that Lfampin resolved 100% of the cases of intestinal amoebiasis, whereas Lfcin 17-30 and Lfcin-B were effective in resolving infection in 80 and 70% of cases, respectively. These data show that although synthetic bovine Lf-derived peptides exhibit varying amoebicidal potentials *in vitro*, they do resolve murine intestinal amoebiasis efficiently, suggesting that they may be useful as a therapeutic treatment.

## Introduction

Amoebiasis caused by the protozoan parasite *Entamoeba histolytica* is ranked as the third leading parasite-associated cause of human mortality worldwide, behind malaria and schistosomiasis [1]. Although the Global Burden of Diseases, Injuries, and Risk Factors Study conducted in 2010 showed that deaths due to amoebiasis have decreased by nearly half in the past two decades, it also stated that amoebiasis is the second leading cause of intestinal parasitosis, just behind cryptosporidiosis [2].

Treatment of amoebiasis relies on the use of imidazole derivatives such as metronidazole, which is highly effective but has the drawback of inducing side effects, being mutagenic at high concentrations and inducing the development of cellular resistance [3]. Amoeba cultures resistant to high concentrations of metronidazole have been obtained *in vitro* via continuous exposure to increasing concentrations of metronidazole, and this resistance is associated with an increase in superoxide dismutase activity with no changes in pyruvate:ferredoxin oxidoreductase activity [4]. This suggests the possible existence of circulating amoebic populations that have developed resistance to metronidazole, reducing the efficacy of the treatment of choice. The identification of new compounds that possess potent anti-amoebic activity and are safe for use in humans has therefore become a priority in the field of amoebiasis treatment.

A viable possibility is the use of lactoferrin (Lf), a conserved iron-binding mammalian glycoprotein with potent antimicrobial activity present in secretions of mucosal surfaces, which are regarded as portals of entry and/or invasion of pathogens [5]. Lf is considered to be a molecule involved in the mammal innate-immune defense and exerts antimicrobial activity against nearly any pathogen [6]. Mechanisms underlying the antimicrobial action of Lf result from microbiostatic and/or microbicidal, as well as immunomodulatory, effects [7–9]. This latter feature enables Lf to escape the mechanisms of direct microbial resistance by acting more on the modulation of an optimal immune response, rather than directly on the pathogen. When ingested, Lf cleavage generates several peptides from the N-terminal region called lactoferricins (Lfcin), which usually exhibit more potent anti-bacterial activity than the native protein [10,11]. A 14-residue peptide from amino acids 17 to 30 (Lfcin 17-30) is obtained after cleavage of bovine Lf with pepsin, whereas a 11-residue peptide from amino acids 4 to 14 (Lfcin-B) is obtained from calf rennet hydrolyzed Lf. Both peptides show potent broad-spectrum antibacterial activity much higher than that of Lf [10,12]. In addition, a peptide from the C-terminus (amino acids 265–284) called lactoferrampin (Lfampin) has also been identified as possessing bactericidal [13] and candidacidal [14] activities. As with Lf, the Lf-derived peptides are also capable of killing pathogens by disturbing their cell membranes, as well as exerting immunomodulatory effects [9,15]. Thus, Lf-derived peptides are a promising class of antimicrobial compounds for fighting pathogenic microbes.

In previous studies, we have reported the amoebicidal effects of Lf and the Lf-derived peptides Lfcin-B, Lfcin 17-30, and Lfampin, as well as the chimera Lfcin 17-30/Lfampin, on cultures of *E. histolytica* trophozoites [16,17]. In this work, we analyzed in greater detail the *in vitro* amoebicidal effect of the three peptides, demonstrated that only Lfampin significatly affect amoeba viability even when all peptides were internalized by the parasites using the same endocytic route, identified possible targets in the amoeba based on docking studies and showed that these three peptides can resolve an established amoebic intracecal infection in C3H/HeJ mice.

## Materials and methods

### Peptides

Unlabeled and fluorescein isothiocyanate (FITC)-labeled bovine Lf peptides, Lfcin-B (RRWQWRMKKLG), Lfcin 17-30 (FKCRRWQWRMKKLG), and Lfampin (DLIWKLLSKAQEKFGKNKSR) were synthesized in the GenicBio Limited Laboratory of the Chinese ChemNet Global Chemical Network company. Peptide stocks were prepared in phosphate-buffered saline (PBS).

### *Entamoeba histolytica* cultures

Axenic *E. histolytica* HM1:IMSS trophozoites were maintained in TYI-S-33 medium supplemented with 15% adult bovine serum (Microlab) and 3% Diamond vitamin stock solution 40× (Sigma-Aldrich) under anaerobic conditions at 37°C for 72 h. Virulence of trophozoites was maintained through successive passages into hamster liver, followed by recovery of parasites from the induced amoebic liver abscesses. Trophozoites grown for 72 h (log-phase) were harvested by ice-chilling for 5 min and centrifugation at 150 ***g*** for 5 min at 4°C.

### Viability assays

Trophozoites were harvested from cultures after 72 h, as mentioned above, and were seeded in 96-well culture plates at 2 × 10^4^ trophozoites in 200 μl of fresh TYI-S-33 medium. Immediately, 10 µl of PBS alone (control) or 10 μl of PBS containing 1, 10, 100, 250, 500, or 1000 µM of Lfcin-B, Lfcin 17-30, or Lfampin peptides were added to each well. Assays using the three peptides combined at concentrations of 1, 10, and 100 µM were also carried out. The plates were incubated for 24 h at 37°C and viability was determined using 3-[4,5-dimethylthiazol-2-yl]-2,5-diphenyltetrazolium bromide (MTT). In brief, 80 μl of MTT (1 mg/ml) were added to each well and the plates were incubated for 1 h at 37°C in darkness. Afterwards, the plates were centrifuged at 1400 rpm for 5 min and supernatant was removed to preserve a volume of 100 μl per well. Each well received 100 μl of 60°C sodium dodecyl sulphate:hydrochloric (15% SDS, 0.01 N HCl) and the contents of wells were then homogenized to dissolve formazan crystals that formed. Finally, the absorbance at 595 nm was measured within 15 min, using a plate reader Multiskan FC (Thermo Scientific). A MTT calibration curve was previously calculated using amoeba densities of between 1 × 10^3^ and 1 × 10^5^ parasites/well. Trophozoites killed via treatment with 0.1, 1, 5, 10, or 15% of dimethyl sulfoxide (DMSO) were used to evaluate the sensitivity of the assay to determine cell death. The linearity of the assay was demonstrated after five repetitions (*r* = 098). All experiments were performed in three independent trials, each one in triplicate. The total number of parasites after treatments was also determined following the same methodology, but adding to the wells only 250 µM of the peptides separately and incubating for 24 h at 37°C. Amoebas were harvested by ice chilling for 5 min and counted on a hemocytometer using a Nikon TMS inverted microscope.

### Apoptosis/necrosis assays

Apoptosis assays were performed using the FITC Annexin V Apoptosis Detection Kit I (BD Pharmingen) following the manufacturer’s instructions. In brief, trophozoites were cultured at 5 × 10^4^ cells per 200 μl of fresh TYI-S-33 medium in 96-well culture plates. Amoebas were treated with 250 µM Lfcin-B, Lfcin 17-30, Lfampin, or PBS (vehicle) for 24 h at 37°C. Treated trophozoites were chilled on ice for 10 min, collected in Eppendorf tubes and washed with PBS. After washing, amoebas cultured during 24 h at 37°C without peptide treatment were further incubated for 30 min at 56°C (death positive control). Binding buffer and Annexin V/propidium iodide (PI) stains were then added to the trophozoites. Finally, the samples were analyzed via flow cytometry in a FACScalibur (Beckton Dickinson). At least 1 × 10^4^ gated events of each sample were analyzed. Non-stained trophozoites were used as the autofluorescence control and heat-treated amoebas (cultured at 56°C during 30 min) were used for compensation. Data were analyzed using FlowJo X software to determine the percentage of viable (PI negative, annexin V-FITC negative), apoptotic (PI negative, annexin V-FITC positive), and necrotic (PI positive, annexin V-FITC positive) cells.

### Interaction of the peptides with trophozoites

To determine the interaction of the peptides with the trophozoites, assays were performed with the three peptides conjugated to FITC. Trophozoites (1 x 10^5^) were seeded in 200 µl of fresh TYI-S-33 medium in 96-well culture plates and treated with 50 µM FITC-labeled peptides for 5 min on ice chilling (bound peptide) or 2 h at 37°C (bound and internalized peptide). After incubation, the trophozoites were chilled on ice for 5 min and transferred into Eppendorf tubes. Amoebas were washed twice with PBS and then fixed with 3.7% formaldehyde for 15 min at room temperature (RT). Finally, the samples were resuspended in 500 µl of PBS and analyzed by flow cytometry in a FACScalibur (Beckton Dickinson). At least 1 × 10^4^ gated events of each sample were analyzed. Non-stained trophozoites were used as the autofluorescence control and results were obtained from three independent trials. Data were analyzed using FlowJo X software for obtaining mean fluorescence intensity values. R1 positive region was determined selecting all values higher than the autofluorescence control. R2 region (binding peptide) was determined selecting 90% of values at 5 min of incubation on ice chilling. R3 region (bound and internalized peptide) was defined as all values higher than R2.

To determine the subcellular localization of the peptides inside amoebas, trophozoites (1 × 10^5^) were cultured in 200 µl of fresh TYI-S-33 medium with Hoechst 33342 (Sigma) (16.23 µM) in 96-well culture plates and treated with 50 µM FITC-labeled peptides for 2 h at 37°C. After incubation, the trophozoites were chilled on ice for 5 min and transferred into Eppendorf tubes. Amoebas were washed twice with PBS and then fixed with 3.7% formaldehyde for 15 min at RT. After fixation, cells were washed with PBS, resuspended in Fluoroshield (Sigma-Aldrich) and analyzed by fluorescence microscopy using a Nikon A1R+ confocal microscope with a magnification lens of 40× and capturing single optical sections. Images were amplified at 90% using NIS elements viewer software (Nikon) and merge was performed using ImageJ software. Results were obtained from three independent trials.

### Entry route of the peptides

To identify the possible route by which the peptides entered the parasites, trophozoites were treated with the three peptides conjugated to FITC, as mentioned above. The fixed trophozoites were then permeabilized for 5 min with 0.2% Triton X-100, washed with PBS, and blocked with bovine serum albumin. Amoebas were incubated with 1:50 diluted mouse anti-clathrin heavy chain, rabbit anti-EEA-1, mouse anti-mannose 6-phosphate receptor or rat anti-LAMP-1 antibodies for 60 min at RT. After extensive washes with PBS, rhodamine-conjugated anti-mouse, TRITC-conjugated anti-rabbit or Alexa Fluor 594-conjugated anti-rat antibodies (diluted 1:50) were respectively added and incubated for 60 min at RT, while protected from light. After several washes with PBS, the trophozoites were incubated for 15 min with DAPI as a nuclear counterstain and washed with PBS. Finally, the trophozoites were resuspended in Fluoroshield (Sigma-Aldrich) and analyzed by fluorescence microscopy using a A1R+ confocal microscope with a lens of 40×. Images were taken capturing single optical sections (Nikon).

To determine whether the internalization of the peptides to the amoeba depends on receptor mediated endocytosis (linked to PI3K signaling) and the actin cytoskeleton, internalization was evaluated by FACs in the presence of specific inhibitors. Briefly, 5 × 10^5^ trophozoites were incubated for 1 h in 500 μl of fresh TYI-S-33 medium added with 100 μM Wortmannin (Calbiochem), 10 μg/ml cytochalasin B (Sigma), or 1% DMSO (vehicle). The amoebae were then treated with 50 μM FITC-labeled peptides for 2 h at 37°C. After incubation, the trophozoites were chilled on ice to stop the internalization of the peptides and washed with cold PBS. The trophozoites were resuspended in 500 μl cold PBS and 2 mg/ml Trypan blue was added before the FACS analysis to quench the external fluorescence. At least 1 × 10^4^ gated events of each sample were analyzed. Non-stained trophozoites were used as autofluorescence control. The results were obtained from three independent trials and the data were analyzed using FlowJo X software.

### Molecular docking

Docking assays were performed using the Autodock v.4.2 [18] and Autodock VINA [19] programs, processing the interactions between Lf-derived peptides and seven homologous structure models, and between Lf-derived peptides and three crystallographic structures of selected ligands from *E. histolytica*. The predicted models were refined in ModRefiner server (http:/ /zhanglab.ccmb.med.umich.edu /ModRefiner/). For all docking experiments, the receptors were kept rigid, enabling the ligands to execute a maximal number of rotatable bonds. All crystallographic water molecules were deleted from the initial structures and the grid size was adjusted to 0.30 Å. The three-peptide Lf molecule was centered as the macromolecule binding site for each ligand. The size and center of the grid box was set to cover the Lf peptide size. Gasteiger charges were added and non-polar hydrogens merged. All docking experiments were carried out in triplicate and *K*_i_ was estimated.

### Infection and treatment protocols

Six-week-old male C3H/HeJ mice (Jackson Laboratory, Bar Harbor, U.S.A.) were maintained in sterile cages with water and food *ad libitum*. Using a protocol approved by the Institutional Animal Care Committee, the mice were divided into four groups of 10 animals each. All mice were intracecally infected with virulent *E. histolytica* trophozoites recovered from amoebic liver abscesses induced in hamsters. In brief, mice were anesthetized with ketamine (90 mg/kg) and xylazine (10 mg/kg), and their abdominal areas were shaved. A laparotomy was conducted under aseptic conditions to expose the cecum and 10^6^ trophozoites in 100 μl PBS were directly injected into its apical zone. The site of injection was immediately obtruded by applying a gel foam pad, the cecum was carefully returned to the peritoneal cavity, and the abdominal layers were sutured (Vycryl 4–0).

Twelve days post-infection, fecal samples were collected from each mouse and processed via ELISA to detect anti-*E. histolytica* IgA antibodies, in order to confirm infection. In brief, fecal samples from day 0 (before infection) and day 15 (post-infection) were thoroughly resuspended in PBS (5 volumes per mg weight) supplemented with a mix of protease inhibitors (Sigma, St. Louis, MO, U.S.A.) and centrifuged at 500 ***g*** at 4°C. Next, 96-well plates were coated overnight with 1 μg/well raw extract (five cycles of thawing/freezing) of *E. histolytica* HM1:IMSS trophozoites in carbonate buffer, pH 9.6, at 4°C. After blocking with BSA-Tween 1%, undiluted fecal supernatants (200 μl) were added and incubated for 1 h at 37°C. After washing with PBS-Tween 20, a goat HRP-conjugated anti-mouse IgA antibody was added to the wells prior to incubation for a further 1 h at 37°C. Finally, after washing, the immune complexes were developed with orthophenylene diamine reagent and absorbance was measured at 490 nm.

Once infection was confirmed, at day 15, mice from groups one, two, and three were treated for 4 days with a daily dose of 10 mg/kg of Lfcin-B, Lfcin 17-30, or Lfampin, respectively. Treatment was given via the oral route in 200 μl PBS using a plastic cannula (standard wall spaghetti tubing; Chemplast Inc., U.S.A.). Mice in group four received only PBS without peptides (Sham control). Two days later (i.e., 20 days post-challenge, time at which the largest amoeba-induced lesions would be expected; [20]), mice were killed with excess of anesthesia. Ceca were excised from all mice, fixed in 10% formaldehyde-PBS and embedded in paraffin. Finally, at least three 5 μm-thick tissue sections at each third of the organ were obtained in a microtome and stained with hematoxylin-eosin stain for histological examination.

### Statistical analysis

Comparison of infection rates between each treatment group and its relevant Sham control was conducted using Fisher’s exact test. Group means and viability data were compared using paired two-tailed Student’s *t*-tests. Data are reported as mean ± SD. A *P*-value ≤ 0.05 was considered statistically significant.

## Results

### Lactoferrin-derived peptides exhibit variable anti-amoebic activity

**Figure 1:**
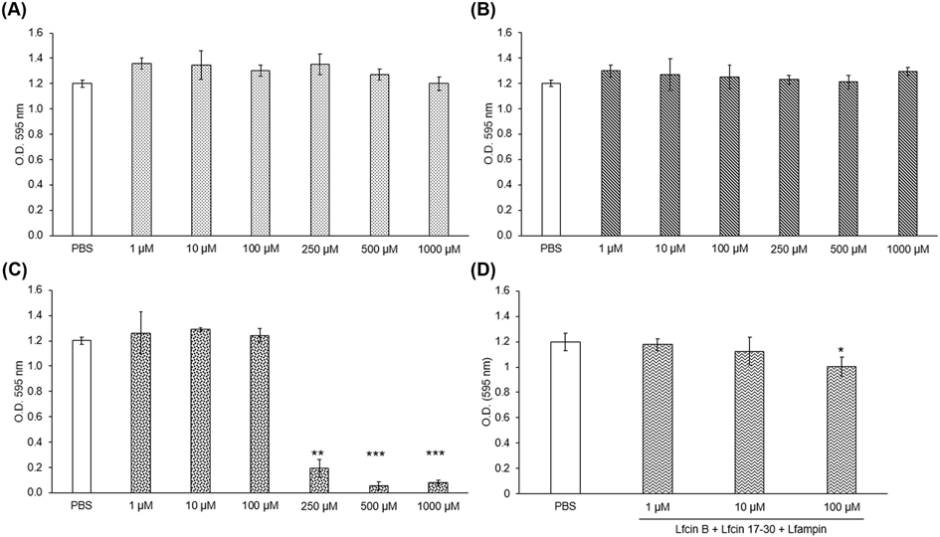
Lactoferrin (Lf)-derived peptides exhibit varying amoebicidal activity Trophozoites (2 × 10^4^) were cultured in fresh TYI-S-33 medium in the presence of (**A**) Lfcin-B, (**B**) Lfcin 17-30, (**C**) Lfampin, or (**D**) the three peptides together at crescent concentrations for 24 h at 37°C. After incubation, cells were centrifuged and the supernatant was removed, preserving 100 μl of medium. MTT solution was added and trophozoites were incubated for 1 h at 37°C. Finally, 60°C SDS–HCl was added to lyse the cells and dissolve formazan crystals. Absorbency was measured at 595 nm within 15 min. Values are presented as means ± SD of three independent experiments. ^*^P < 0.05, ^**^P < 0.01, ^***^P < 0.001.

The MTT data showed variations in the abilities of the peptides to affect the viability of *E. histolytica* trophozoites. Whereas Lfcin-B and Lfcin 17-30 did not affect the amoebic viability at any dose tested in 24 h (Figure 1A,B), treatment of parasite cultures with Lfampin resulted in the death of approximately 80% of trophozoites at 250 μM and more than 90% at 500 μM (Figure 1C). Interestingly, evaluation of the combined effect of the three peptides showed that they do indeed have a slight synergistic effect, causing a drop in viability of approximately 10% at concentrations of 100 μM, respectively (Figure 1D). The IC50 value for Lfampin was estimated in 220.28 μM (Supplementary Figure S1).

To establish if Lfampin exerts an amoebicidal (lysis) or amoebostatic (inhibition of cellular division) effect, parasites were counted after the peptide treatment for 24 h at 250 μM. It was observed that Lfampin significantly decreased trophozoite numbers to a value even lower than that originally seeded (Supplementary Figure S2A). In contrast, Lfcin-B and Lfcin 17-30 did not affect trophozoite numbers in comparison with untreated controls (Supplementary Figure S2A). The reduction in trophozoite numbers caused by Lfampin was associated with a large amount of cellular debris, which was corroborated under a light microscope (Supplementary Figure S2B), suggesting that this peptide lyses the trophozoites and thus exerts an amoebicidal effect.

### Lfampin kills amoebas mainly by necrosis

**Figure 2:**
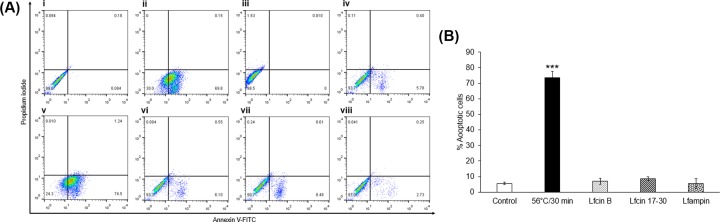
Evaluation of apoptosis and necrosis in trophozoites treated with lactoferrin (Lf)-derived peptides Apoptosis assays were performed using the FITC Annexin V Apoptosis Detection Kit I (BD Pharmigen), following the manufacturer’s instructions. (**A**) In brief, trophozoites (5 × 10^4^) were treated with 250 μM Lfcin-B, Lfcin 17-30, or Lfampin peptides for 24 h. Treated trophozoites were resuspended in binding buffer, to which annexin V/PI stain was added. The samples were analyzed by flow cytometry in a FACScalibur (Beckton Dickinson). (i) Autofluorescence control; (ii) compensation for FITC fluorescence; (iii) compensation for PI fluorescence; (iv) untreated trophozoites; (v) heat-treated trophozoites; (vi) Lfcin-B-treated trophozoites; (vii) Lfcin 17-30-treated trophozoites; and (viii) Lfampin-treated trophozoites. (**B**) Percentage of apoptotic cells (only annexin V positive). ^***^P < 0.01.

As mentioned previously, Lfampin caused a decrease in the viability of amoebas and reduced the number of parasites partly due to necrosis. To determine if Lfampin also induces apoptosis in trophozoites, trophozoites were exposed to this peptide and the others at 250 μM for 24 h and processed by annexin V/PI staining followed by flow cytometry. The results showed that the trophozoites that remained unlysed after treatment with Lfampin did not show phosphatidylserine exposure or incorporation of PI, suggesting that Lfampin kills the amoeba only by lysis (Figure 2A,B). On the other hand, Lfcin-B and Lfcin 17-30 did not induce apoptosis or necrosis in the trophozoites, which agrees with the viability results with MTT (see above).

### Lactoferrin-derived peptides are internalized by *E. histolytica* trophozoites

**Figure 3:**
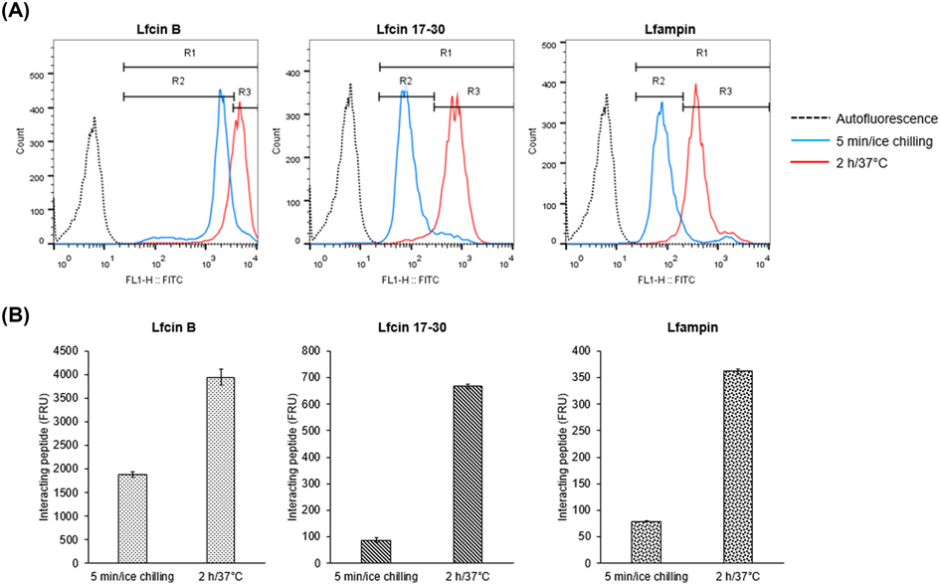
Interaction of lactoferrin (Lf)-derived peptides with *E. histolytica* trophozoites (**A**) Trophozoites (1 × 10^5^) were cultured in 200 μl of fresh TYI-S-33 medium and treated with 50 μM FITC-labeled Lfcin-B, Lfcin 17-30, or Lfampin for 5 min on ice chilling (blue histogram) or 2 h at 37°C (red histogram). After incubation, the trophozoites were washed with PBS and then fixed with formaldehyde. The samples were analyzed by flow cytometry in a FACScalibur. Non-stained trophozoites were used as the autofluorescence control (dotted line histogram). R1 positive region was determined selecting all values higher than the autofluorescence control. R2 region (binding peptide) was determined selecting 90% of values at 5 min of incubation on ice chilling. R3 region (bound and internalized peptide) was defined as all values higher than R2. (**B**) Interacting peptide established as mean intensity fluorescence values at 5 min and 2 h (FRU, fluorescence relative unities). Values are presented as means ± SD of three independent experiments.

**Figure 4:**
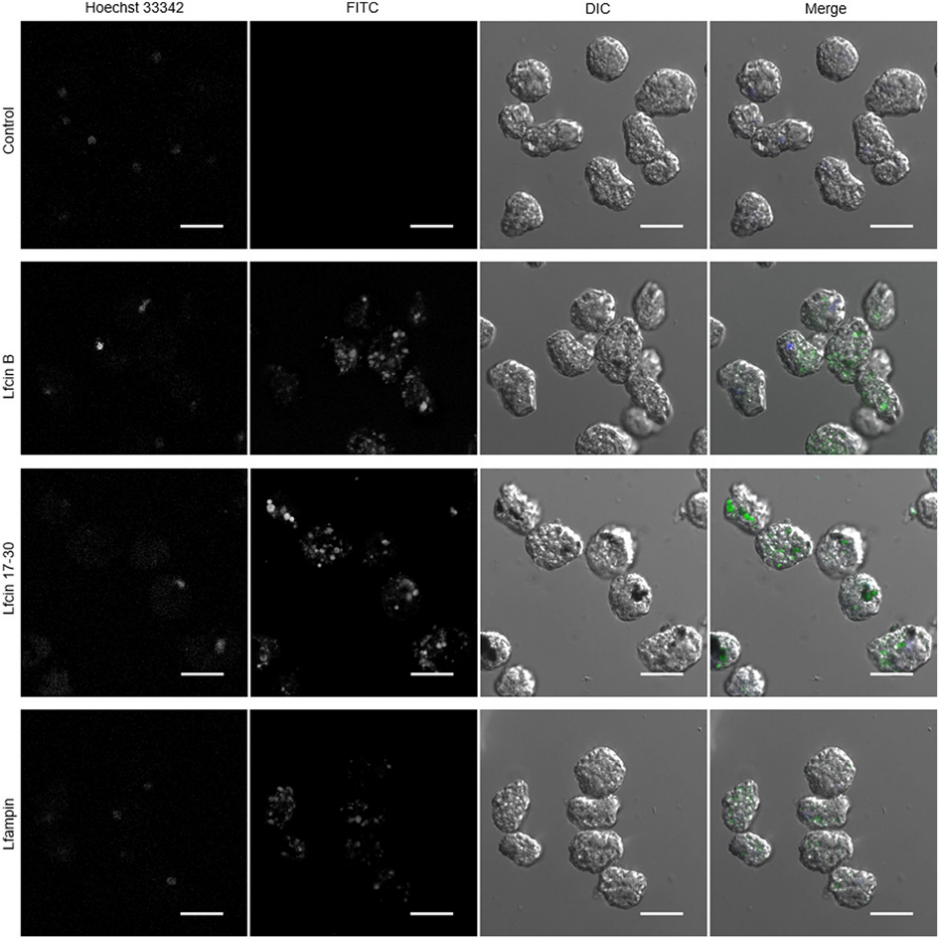
*E. histolytica* trophozoites internalize lactoferrin (Lf)-derived peptides Trophozoites (1 × 10^5^) were cultured in 200 μl of fresh TYI-S-33 medium with Hoechst and treated with 50 μM FITC-labeled peptides for 2 h at 37°C. After incubation, the trophozoites were washed with PBS and fixed with formaldehyde. Amoebas were visualized by fluorescence microscopy using a Nikon A1R+ confocal microscope. Scale bar = 20 μm.

FITC-conjugated Lf-derived peptides were used to determine if amoebas interact with the peptides, if they are internalized by the trophozoites and their subcellular location. Results of flow cytometry showed that FITC fluorescence was displaced at 5 min of incubation of trophozoites with the peptides on ice (Figure 3A). This suggests that the three peptides interact rapidly with the trophozoite’s surface. After 2 h of incubation, fluorescence intensity values increased with respect to the fluorescence at 5 min, suggesting a higher interaction of peptides with trophozoites and probably internalization (Figure 3A,B). Confocal fluorescence microscopy showed that all three peptides were internalized by the trophozoites, being mostly distributed in the cytosol inside large vacuoles and small vesicles. (Figure 4). Colocalization with Hoechst was not observed, suggesting that the peptides did not interact with nuclei after 2 h of exposure. Fluorescence was also not associated with the plasma membrane, suggesting that most of the peptides were internalized after interaction with amoebas (Figure 4).

**Figure 5:**
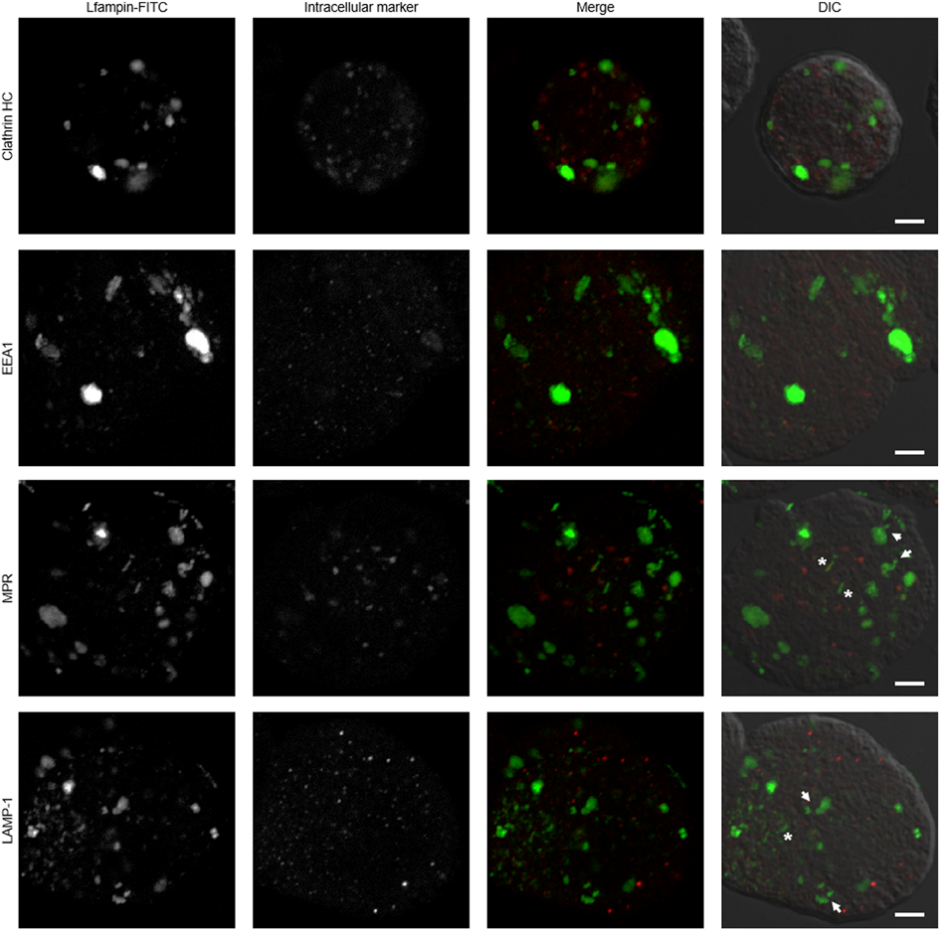
Internalization pathway of FITC-coupled lactoferrin-derived Lfampin into *E. histolytica* trophozoites Amoebas (1 × 10^5^) were cultured in fresh TYI-S-33 medium in the presence of 50 μM FITC-labeled Lfampin for 2 h at 37°C. Cells were fixed and immunofluorescence was performing using anti-clathrin HC, anti-EEA1, anti-MPR, and anti-LAMP-1 antibodies, followed by anti-mouse IgG conjugated to rhodamine (for clathrin HC and MPR), anti-rat IgG conjugated to Alexa Fluor 594 (for LAMP-1), or anti-rabbit conjugated to TRITC (for EEA1). White arrows indicate vesicles that appear to interact with vacuoles and asterisks indicate tubular-like structures. Scale bar = 5 μm.

As previous studies have demonstrated that lactoferrin enters amoebas through endosomes [16], in this work, the co-localization of all three peptides with clathrin-coated vesicles, early endosomes, late endosomes, and phagolysosomes at 2 h of exposure was evaluated to establish differences that could help explain the amoebicidal activity of Lfampin. FITC-labeled Lfampin was detected in the cytosol of amoebas in multiple small vesicles and in a few very large asymmetric and non-uniform vacuoles (Figure 5). In a few cases, the mark was observed in vesicles that appeared to interact with the vacuoles as if they were being integrated into them (Figure 5, white arrows) and into independent, elongated, tubular-like structures (Figure 5, asterisks). Nevertheless, FITC-labeled Lfampin did not co-localize with clathrin, early endosomes, late endosomes, or lysosomes (Figure 5), suggesting an internalization mechanism independent of the clathrin-endosomal route. Except for the asymmetric and non-uniform vacuoles with Lfampin, similar results were obtained with the FITC-labeled Lfcin-B and Lfcin 17-30 peptides (data not shown).

**Figure 6:**
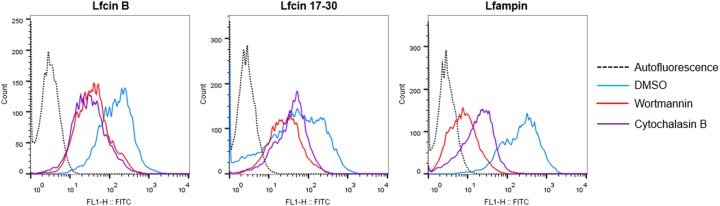
Internalization of lactoferrin-derived peptides depends on receptor-mediated endocytosis and actin cytoskeleton dynamics Trophozoites (5 × 10^5^) were incubated for 1 h in TYI-S-33 medium added with Wortmannin (100 µM), cytochalasin B (10 µg/ml), or 1% DMSO (vehicle). Amoebas were then incubated with 50 µM FITC-labeled peptides for 2 h at 37°C. After incubation, cells were washed with cold PBS. Trophozoites were resuspended in cold PBS and 2 mg/ml Trypan blue was added. The samples were analyzed by flow cytometry in a FACScalibur.

FACs analysis was performed to determine the dependency of peptide internalization of PI3K signaling (linked to receptor-mediated endocytosis) and of the actin cytoskeleton dynamics. It was observed that treatment of trophozoites with wortmannin or cytochalasin B reduced the internalization of Lfcin-B, Lfcin 17-30, and Lfampin peptides compared with DMSO-treated trophozoites (Figure 6). The inhibitors, however, did not completely block the entry of peptides, since fluorescence was observed in the trophozoites treated with the inhibitors when Trypan blue was used, which was able to quench all extracellular fluorescence (data not shown). These results suggest that mechanisms dependent on receptor-mediated endocytosis and the actin cytoskeleton participate in the internalization of the peptides to the amoeba but are not the only ones involved. In the case of fluorescence by Lfampin, however, wortmannin seems to reduce fluorescence more than cytochalasin B (Figure 6), suggesting that an internalization pathway that requires PI3K signaling might be more important than the actin cytoskeleton for the internalization of this peptide to the trophozoites. In the case of peptides LFcin-B and Lfcin 17-30, the fluorescence is reduced equally by both inhibitors (Figure 6).

### Molecular docking predicts that Lf peptides bind to several amoebic proteins and cholesterol

**Figure 7:**
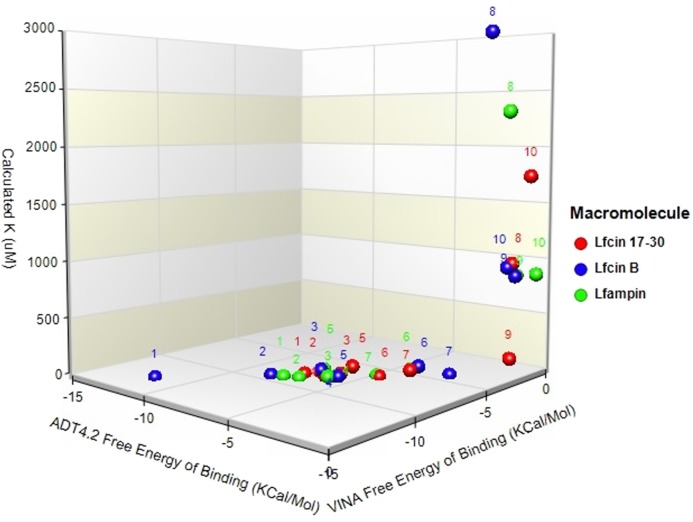
Clustering of free energy of binding vs calculated *K*_i_ for lactoferrin (Lf)-derived peptides docked to *E. histolytica* molecules and cholesterol Molecular dataset of hierarchical clustering plot from two different docking calculation datasets against the peptides. One subset of molecules (red dots) displayed belong to Lfcin 17-30, the second subset (blue dots) to Lfcin-B, and the third subset (green dots) to Lfampin. Numbers on the plot correspond to the following molecules in order of best docking scores: (1) deoxyuridine 5-triphosphate nucleotidohydrolase (3LQW), (2) Protein G (4FID), (3) cholesterol (5997), (4) triplet AAA, (5) clathrin B (10588140), (6) EhCBP1 (5XOP), (7) phenylkainic acid (10933893), (8) Dbr1 (5K73), (9) HP1 (2M2L), and (10) α-actinin-1 (2M7L).

Molecular docking was conducted on 25 molecules, from which nine positive predictions were found to exhibit potential binding: Protein G (4FID), deoxyuridine 5-triphosphate nucleotidohydrolase (3LQW), clathrin (10588140), EhCaBP1 (5XOP), Dbr1 (5K73), HP1 (2M2L), αB-actinin-1 (2M7L), phenylkainic acid (10933893), and cholesterol (5997). These structures showed favorable binding found in an *in silico* prediction. For each molecule, many docking poses were executed to find the highest docking score with a favorable hydrogen bond interaction. The best docking scores are shown in Table 1. As a positive control, we used a triplet of adenine because there is strong evidence that Lf binds to DNA under stringent acidic conditions and this method has proved best over a broad variety of techniques [21]. At the top of our list of predictions is Protein G; however, the top five were in the same range as or above the DNA score that has been experimentally proved as a ligand of Lf (Figure 7).

**Table 1:** Chart depicts the best matches between *E. histolytica* molecular structures and LF peptides in descendent order of prediction value

Molecule structure set	Code	VINA	ADT4.2	Calculated *K*_i_ (µM)
		Free energy of binding (Kcal/mol)	Free energy of binding (Kcal/mol)	Lf17-30/ampin/B
		Lf 17-30/ampin/B	Lf 17-30/ampin/B	
Protein G	4FID	−7.32/−8.21/9.7	−11.85/−12.92/2.1	−7.76/−9.10/4.8
DTN	3LQW	−6.79/−7.68/5.52	−8.29/−9.32/7.92	−7.09/−8.81/2.15
Cholesterol	5997	−5.92/−7.41/25	−6.39/−8.15/72	−5.91/−8.28/31
Adenine triplet	190	−6.14/−8.12/15	−5.93/−7.92/6.2	−5.98/−7.64/6.2
Clathrin B	10588140	−5.80/−6.72/68	−5.64/−7.89/27.8	−6.92/−7.35/28.2
EhCBP1	5XOP	−4.65 /−6.30/2	−3.59/ −4.85/74	−3.97/−4.87/35
Phenylkainic acid	10933893	−3.52/−5.56/67.20	−2.46/−4.09/9.3	−4.80/−6.28/4.2
Dbr1	5K73	−0.48/−2.29/9989	−1.26/−2.88/2987	−0.53/−2.54/2309
HP1	2M2L	−0.47/−2.42/153	−0.54/−1.94/870	−1.34/−0.43/829
α-Actinin-1	2M7L	−0.12/−1.42/1746	−0.61/−2.49/956	−0.25/−0.59/862

### Lactoferrin peptides resolve intracecal infection with *E. histolytica* trophozoites

**Figure 8:**
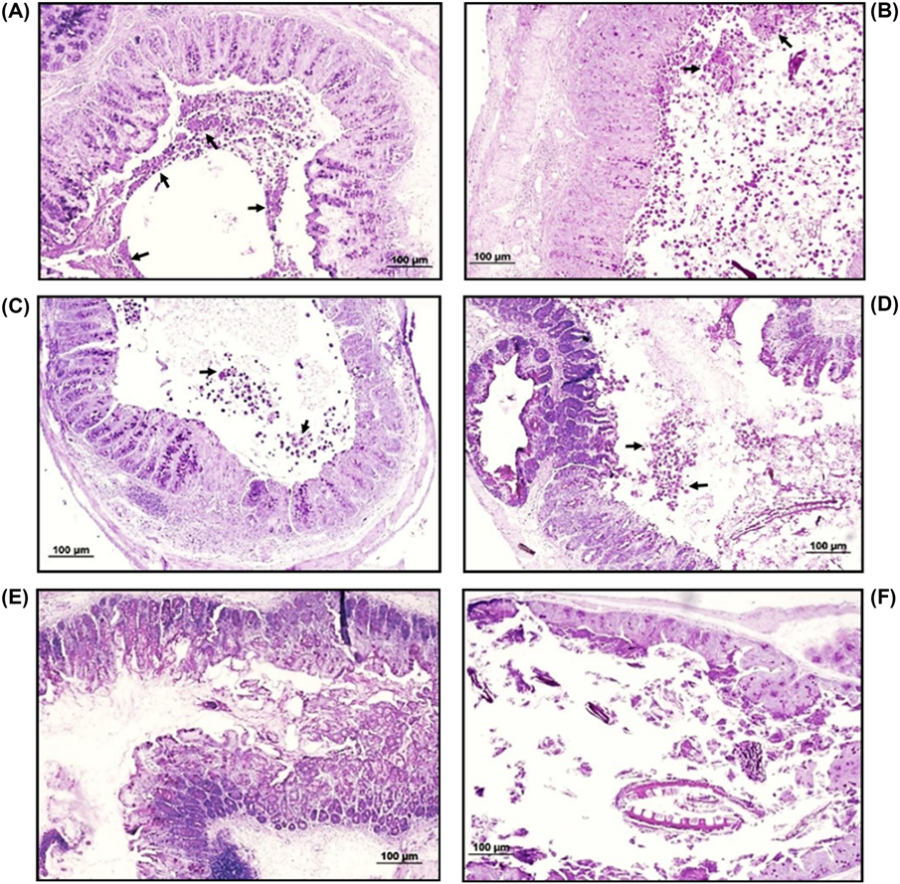
Representative images of cecal tissue sections from infected mice treated with lactoferrin (Lf)-derived peptides C3H/HeJ mice intracecally infected with *E. histolytica* trophozoites were treated at 15 days post-infection via the oral route with 10 mg/kg of each peptide for 4 days. Animals were killed 2 days later and ceca were processed for histopathological analysis. (**A** and **B**) Tissue sections from Sham mice showing abundant amoebas in the intestinal lumen, with erosion of the enteric epithelia. (**C** and **D**) Tissue sections from Lfcin 17-30- and Lfcin-B-treated mice, respectively, showing reduction in the load of amoebas contained in the intestinal lumen. (**E** and **F**) Tissue sections from Lfampin-treated mice showing complete absence of amoebas in the intestinal lumen, indicating eradication of the infection. H&E staining. Arrows show amoebas in intestinal lumen. Scale bar = 100 μm.

To determine the ability of the three peptides to control intestinal amoebiasis, C3H/HeJ mice infected with the parasite at day 15 post-infection (confirmed by the presence of anti-amoeba IgA antibodies in feces by ELISA; data not shown) were orally treated daily for 4 days with 10 mg/kg of each peptide separately. The dose of peptide used was based in our previous study showing that lactoferrin resolved murine intracecal amoebiasis [22]. Upon murine sacrifice, ceca were excised from the abdominal cavity and H&E tissue sections from those tissues were examined under microscope. Data from two independent protection experiments with five animals per group revealed clear differences in infection rates between mice in the Sham group (PBS-treated) and the other three Lf-derived peptide-treated groups. Thus, all 10 mice from the Sham group showed cecal infection with a large number of trophozoites and abundant mucus in the lumen (Figure 8A,B, arrows). In contrast, mice in the groups treated with Lfcin 17-30 or Lfcin-B showed an absence of amoebic trophozoites in the lumen of 75% of the animals or a decrease in parasitic load (approximately two-thirds) in the remaining 25% of the mice (Figure 8C,D; Table 2). Moreover, treatment with Lfampin eradicated the infection in 100% of the animals (Figure 8E,F; Table 2), demonstrating that it is more active as an anti-amoebic agent than are Lfcin 17-30 and Lfcin-B.

**Table 2:** Resolved and reduced infection rates by oral treatment with Lf peptides upon mice with an intracecal amoebic infection previously established

Groups	Resolved infection rate (%)	Reduced parasite load rate (%)	Trophozoite count/mm^2^	*p*-Value by Fisher’s test
1: LFcin 17-30	8/10 (80%)	2/10 (20%)	133 ± 27	0.0007 (vs Sham)
2: LFcin B	7/10 (70%)	3/10 (30%)	117 ± 32	0.0031 (vs Sham)
3: LFampin	10/10 (100%)	N.A.	0	0 (vs Sham) NS (vs Lfcins)
4: PBS (Sham)	0/10 (0%)	0/10 (0%)	392 ± 46	NA

## Discussion

Intestinal parasite infections, including amoebiasis, remain the main causes of morbidity and mortality in developing countries [2]. In Mexico, amoebiasis is included in the list of the 20 most frequent communicable diseases, as the numbers of new cases of intestinal and hepatic amoebiasis in 2016 were approximately 220000 and 560, respectively, according to the Secretary of Health (http://www.epidemiologia.salud.gob.mx/anuario/2016/morbilidad/nacional/distribucion_casos_nuevos_enfermedad_fuente_notificacion.pdf). Treatment with metronidazole and other nitro-imidazole derivatives remains the main strategy for controlling amoebiasis, but their drawbacks, which often lead to abandonment of the therapy, in addition to the potential development of resistance by the parasite [23–25], highlight the need to identify new highly effective and less toxic anti-amoebic compounds. One of the most promising options is the use of molecules of natural origin with broad anti-microbial potential, such as lactoferrin, a breast milk protein considered part of the innate immunity of mammals. In a previous study conducted by our group, we demonstrated that lactoferrin exerts a potent anti-amoebic effect *in vitro* and *in vivo*, killing parasites in culture and resolving intestinal infection in a murine model by up to 63% with a daily regimen of 20 mg/kg orally for 7 days [22]. More recently, peptides derived from Lf have become important as antimicrobials due to their great effectiveness (including greater activity than Lf in cases of resistant bacteria) and the advantages offered by synthesizing them [26]. In addition to being synthesized in large quantity, synthetic peptides are more stable than Lf in relation to temperature and humidity changes and eliminate the potential risk of biological contamination that can occur when Lf is purified from milk. Although many antimicrobial peptides from lactoferrin have been characterized, only Lfcin-B, Lfcin 17-30, and Lfampin have been studied in detail. A previous study reported that Lfcin 17-30 and Lfampin, as well as the chimera Lfcin17-30/Lfampin, exhibit *in vitro* anti-amoebic activity [17]. The mechanism of amoebicidal action of the peptides, the type of death they induce and their potential to resolve an intestinal infection *in vivo* remain unknown, however.

Herein we show that under our conditions and using the MTT technique to evaluate the viability of the amoebas, the peptide Lfampin is able to kill trophozoites at concentrations greater than 100 µM. Whereas Lfampin kills amoebas by necrosis in a short period of time (24 h) and in a dose-dependent manner, the Lfcins do not kill these parasites at any dose evaluated at 24 h post-infection. Our results are consistent with a previous study that showed that Lfampin kills amoebas *in vitro*, although that study differed from ours regarding the concentration of Lfampin that killed amoebas. The previous study mentioned that Lfampin kills approximately 50% of *E. histolytica* trophozoites in 24 h at 50 µM [17]. That study reported the same result for Lfcin 17-30 [17]; in our case, however, we found that this peptide does not have amoebicidal activity. The reason for this discrepancy is unknown, but it could be related to differences in the methods used for evaluating viability (MTT vs Trypan blue). The determination of amoebic death by Trypan blue exclusion can lead to errors in quantification, as we note that live amoebas can incorporate aggregates of blue-stained peptides, which can be confused with incorporation due to membrane damage. Another possibility is variation in susceptibility of the *E. histolytica* strain HM1:IMSS to this peptide, due to the frequency with which amoebas are inoculated into the liver of hamsters to maintain virulence. Our amoebic cultures were kept virulent by monthly passes, but the virulence of the cultures used in the previous study is not indicated. The *in vitro* effectiveness of Lfampin to kill other protozoa, such as *Leishmania donovani* [27] or *Giardia intestinalis* [28], has also been reported, supporting the possibility of using it alone or in combination with the usual therapies as a therapeutic agent for the common treatment of several parasitic infections that afflict humans, including concomitant infections.

Because not all amoebas detected as killed by MTT (∼80% at 250 μM) were lysed by the Lfampin peptide, we evaluated if this peptide was capable of inducing apoptosis. In fact, apoptosis in *E. histolytica* trophozoites has been reported by exposure to compounds such as gentamicin or resveratrol, which induce high levels of reactive oxygen species [29,30]. The flow cytometry analysis showed that non-lysed amoebas were still viable, with no signs of apoptosis or necrosis. In agreement with the MTT results, Lfcin-B and Lfcin 17-30 did not induce apoptosis or necrosis in the parasite at 250 μm after 24 h.

Binding and internalization assays of labeled peptides showed that all three peptides were rapidly bound to the trophozoite’s membrane (5 min) and then internalized by the trophozoites mainly via a route independent of clathrin-mediated endocytosis but dependent on receptor-mediated endocytosis (PI3K signaling) and actin cytoskeleton dynamics. This contrasts with reports of internalization into amoebas of holo-lactoferrin and other iron-binding proteins such as transferrin and ferritin, which have been reported to be taken into amoebas through clathrin-coated vesicles [31,32]. On the other hand, the inhibitors did not completely block the entry of peptides, suggesting that other mechanisms are also involved. In this regard, the ability of certain peptides to translocate the plasma membrane and enter directly into cells has been described. The characteristics of such peptides include being short, partly hydrophobic and having a net positive charge at physiological pH, characteristics that are shared by Lf-derived peptides (reviewed in [33]). Conversely, the incorporation of small molecules such as peptides by the amoeba through pinocytosis and macropinocytosis is another possibility [34,35]. Events of clathrin-independent endocytosis and coupling of this to processes such as macropinocytosis by the acute remodeling of the membrane have recently been described (reviewed in [36]). The process has been described in a broad range of organisms, including yeast, and participates in the bulk of extracellular protein and lipid uptake, among other functions. Of note, clathrin-independent uptake allows the formation of large vesicles with widely different morphologies (including tubular structures) that resemble those we observed in amoebas treated with lactoferrin peptides, mainly with Lfampin, suggesting that the peptides could have entered the parasite via this route. Noteworthy, the results of internalization using inhibitors, suggest that mechanisms dependent on receptor-mediated endocytosis and the actin cytoskeleton are involved in the internalization of the three peptides to the amoeba, of which, receptor-mediated endocytosis seems to be more important for the internalization of Lfampin. If this has to do with its lytic capacity is unknown and will be a subject of study for future trials. In addition, more studies are required to confirm that clathrin-independent pathway exists in amoebas, and whether it participates in the uptake of the peptides or the peptides enter directly into the parasite.

The lytic capacity of Lfampin was evidenced at 24 h by the reduction in the number of amoebas, as well as by the presence of a large amount of detritus (Supplementary Figure S2). The events that lead to necrosis following the internalization of Lfampin are unknown. The complete lysis of amoebas by Lfampin suggests that the peptide induces a toxic stimulus on the trophozoites that overwhelms their homeostatic balance, committing the cells to an uncontrolled death by likely disturbing the hydrophobic interphase of the lipid bilayer. This result agrees with others suggesting that Lfampin plays key role activities by exerting its interaction with cellular membranes, thanks to the positive charge of its C-terminal end and the helical structure of its amino-terminal [13,14]. A model concerning the interaction of Lfampin with cell membranes has been proposed [37] and could explain the amoebicidal effect that we observed with this peptide. In this regard, our molecular docking studies predict that Lfampin can bind several amoebic or host proteins with higher affinity than Lfcin 17-30 or Lfcin-B. Of these amoebic or host proteins, the G protein is at the top of our prediction list and although it has been described as being involved in the signaling process related to engulfment in *E. histolytica*, the mechanism is far from being fully described [38]. Deoxyuridine 5-triphosphate nucleotide hydroxide is the second-best prediction but, to our knowledge, there is no information reported regarding any role or conceivable function in *E. histolytica* that could explain the effect of Lfampin on the parasitic membrane. In contrast, high-affinity binding of Lfampin to the trophozoite membranes through cholesterol, the third-best prediction, could explain in part the mechanism by which Lfampin displays its biological effect. Thus, Lfampin binding with high affinity to the amoebic plasma and intracellular membranes through interaction with cholesterol may cause loss of membrane integrity, leading to lysis. This hypothesis is indirectly supported by our previous report showing the presence of cholesterol-dependent endocytosis of apo-lactoferrin in virulent *E. histolytica* trophozoites [32]. Studies using inhibitors of the interaction of the peptide with cholesterol, by competition or blockade, as well as a more detailed analysis of the integrity of the parasitic membranes in the presence of the peptide, are necessary to demonstrate our hypothesis, however.

Finally, our results regarding the high efficiency of the peptides at resolving murine intestinal amoebiasis opens the possibility of using them as potential new compounds for the treatment of the disease, either alone or in combination with metronidazole. This would reduce the concentration of the drug needed to resolve an amoebic infection and, in turn, reduce the adverse effects of the drug and the potential development of resistance in the parasite. The mechanism by which the peptides controlled intestinal infection by amoebas in mice remains unknown. The higher efficiency of Lfampin, which resolved 100% of infected cases in comparison with treated trophozoites the Lfcins (70–80%) or the native Lf (63%) [22], could be due to its superior property of interacting and altering the balance of cellular lipid membranes [37]. We have not yet shown, however, that orally administered peptides reach the surface of amoebas in the cecum of infected mice, so studies administering the labeled peptides and looking for them in the intestine in intimate contact with trophozoites are necessary to demonstrate that they kill amoebas *in situ*. Another possibility is that the peptides help to resolve intestinal infection due to their immunomodulatory properties [9,15], so it is necessary in future studies to evaluate anti-*E. histolytica* immune response in cured animals. In addition, further studies are necessary to determine the amoebicidal ability of the combined three peptides, which could lead to the use of much smaller concentrations, as well as the possibility of adding these peptides to the standard metronidazole treatment. Because they originated from a protein belonging to the innate immune response of mammals, the Lf-derived peptides are suggested to be safe for administration in humans, which constitutes an advantage when proposing them as therapy to treat amoebiasis and other microbial infections. Moreover, Lfcin 17-30 has been shown to be non-toxic in animals and is part of the daily diet of humans, as it is ingested through dairy products or naturally synthesized within the body. Together with native Lf, the Lf-derived peptides have been proposed as a possible treatment against parasitic diseases in humans and as a veterinary drug [39]. More detailed studies of cytotoxicity with the peptides are required, however, mainly with the potentially lytic Lfampin.

In conclusion, our study showed that the lactoferrin-derived peptide Lfampin exhibits moderate amoebicidal activity *in vitro*, but shows potent therapeutic effects via the oral route, completely resolving intestinal amoebic infection in mice. It is worth noting that even though the other peptides, Lfcin-B and Lfcin 17-30, did not affect the viability of *E. histolytica* trophozoites *in vitro*, they also resolved or decreased the parasite load in mice, suggesting that another mechanism not related with amoebicidal activity, likely an immunomodulatory effect, may be controlling the infection. This opens the possibility of using lactoferrin-derived peptides as therapeutic agents for the treatment of amoebiasis in humans.

## Supporting information

**supplementary Figure 1:** 

**supplementary Figure 2:** 
