# Supplementary figures and images for: Synthetic bovine lactoferrin peptide Lfampin kills Entamoeba histolytica trophozoites by necrosis and resolves amoebic intracecal infection in mice
Source: Biosci Rep. 2019 Jan 8;39(1):BSR20180850. doi: 10.1042/BSR20180850 (PMC6328891; doi:10.1042/BSR20180850)

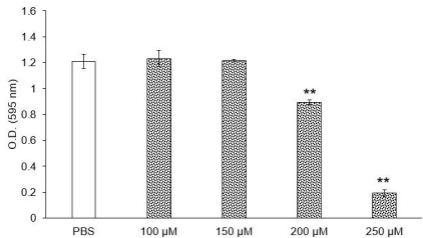

**A**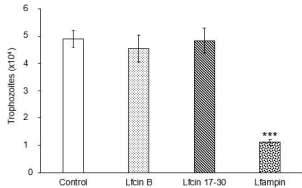**B**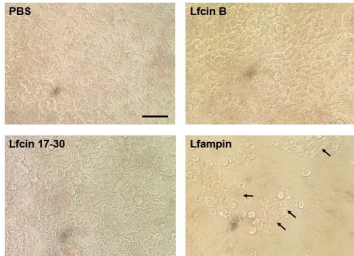

Supplement: Supplementary file 1 [file bsr20180850_Supp1.pdf]
